# Human CD117 (cKit)+ Innate Lymphoid Cells Have a Discrete Transcriptional Profile at Homeostasis and Are Expanded during Filarial Infection

**DOI:** 10.1371/journal.pone.0108649

**Published:** 2014-09-25

**Authors:** Alexis Boyd, José M. C. Ribeiro, Thomas B. Nutman

**Affiliations:** 1 Laboratory of Parasitic Diseases, National Institute of Allergy and Infectious Diseases, National Institutes of Health, Bethesda, Maryland, United States of America; 2 Laboratory of Malaria and Vector Research, National Institute of Allergy and Infectious Diseases, National Institutes of Health, Bethesda, Maryland, United States of America; Beth Israel Deaconess Medical Center, Harvard Medical School, United States of America

## Abstract

Since innate lymphoid cells (ILCs) have been found to play a role in the immune response to helminth parasites in rodents, we sought to determine their role in human helminth infection. By developing multicolor flow cytometry-based methods to identify and enumerate circulating ILCs and their subsets, we were able to identify a subset of cKit+ ILCs defined as Lineage (Lin)−/CD45+/cKit+/CD127+ that were significantly expanded in the filarial-infected individuals (p = 0.0473) as were those cKit+ ILCs that produced IL-13. Additionally, the frequency of these cKit+ ILCs correlated with the frequency of IL-17 producing CD4+ T cells (Th17 cells; p = 0.025). To investigate the function of cKit+ ILCs, sorted, highly purified human ILCs were subjected to transcriptional profiling by RNAseq and compared to appropriate control cells. These cKit+ ILCs expressed TLRs, a broad range of cytokines/cytokine receptors and MHC Class II molecules suggesting that these ILCs sense pathogens independent of other cell types. Functional analysis revealed expanded cKit+ ILC-specific transcription and ILC-specific microRNA precursors.

## Introduction

Innate lymphoid cells (ILCs) are identified by their lack of cell lineage markers associated with T cells, B cells, dendritic cells, monocyte/macrophages, and granulocytes, and their expression of CD127 (IL-7Rα), among others [1]–[4]. It is now known that there are 3 major subsets of ILCs, termed ILC1, ILC2 and ILC3, that each have specific cytokine profiles driven by discrete transcription factors [5]. ILC1s have been shown to produce IL-12 primarily and rely on the transcription factor Tbet; ILC2s produce IL-13, IL-5 and some IL-4 and their differentiation is driven by GATA3; and ILC3s express Rorγt and produce IL-22 and IL-17. ILC subsets can also be identified by expression of particular surface markers, with ILC2s and ILC3s expressing cKit (or CD117) and ILC2s expressing ST2 (IL-33R) and CRTH2, for example [5]. These ILC subsets parallel the subsetting seen among CD4+ T cells and are thought to influence the differentiation of naïve CD4+ T cells into various helper cell subpopulations [5].

ILCs, specifically ILC2s in mice, respond to IL-25 and IL-33 produced from barrier-associated cells by making IL-13 and IL-5 and, to a lesser extent IL-4, which in turn drive a Th2 response [1]–[3]. This family of innate cells has also been identified in human tissues and peripheral blood [6]. Human ILCs have been found at inflammatory sites such as the nasal tissue in rhinosinusitis [6], the gastrointestinal tract in Crohn’s disease [7] and the skin in atopic dermatitis [8]. ILCs have not yet been evaluated either in the context of tissue invasive helminths nor in other human parasitic infections.

Immune responses to helminth parasites in general have been broadly suggested to have a predominant Th2 response that includes an expansion of CD4+ T cells producing a combination of cytokines (IL-5, IL-4, and/or IL-13), the production of IgE antibody and tissue or peripheral blood eosinophilia [9]–[11]. Although filarial infections in humans induce responses associated with a Th2 response, recent studies have revealed that at homeostasis single producing IL-4+, IL-10+ and IL-17+ CD4+ cells are expanded in human filarial infections [12]. However, the innate cells and pathways responsible for facilitating this expansion remain to be fully elucidated.

In the present study, we demonstrate that in filarial infections, caused by a major set of tissue invasive helminth parasites, cKit+ ILCs (comprised of ILC2s and ILC3s) are expanded and this expansion is associated with a concomitant (and parallel) increase in IL-17 producing CD4+ T cells. Through RNA-seq based transcriptional profiling, we show that these cKit+ ILCs at steady-state in normal uninfected donors allow for pathogen sensing, have chemokine and chemokine receptor expression that limit their egress from intravascular spaces, and are programmed to be anti-apoptotic.

## Materials and Methods

### Study Populations

The study population consisted of 21 filarial-infected patients referred to the NIH for evaluation and potential treatment of their filarial infections and 11 filarial-uninfected blood donors. The filarial-infected group was comprised of 17 patients with *Loa loa*, 3 with *Wuchereria bancrofti* and 1 with *Onchocerca volvulus*. Among the 21, 13 were temporary residents of or travelers to filarial-endemic regions while 8 were indigenous to these same regions. The filarial-infected group consisted of 15 males and 6 females whose age ranged from 25–66 (median 46) years. The filarial-uninfected group came from a subset of donors whose gender distribution was (50%male/50%female) with an age range of 18–65 (median 46) years.

### Ethics Statement

All filarial-infected patients were evaluated by the Clinical Parasitology Section of the Laboratory of Parasitic Diseases under a protocol approved by the Institutional Review Board of the NIAID and registered (NCT00001230) and the diagnosis was parasitologically-proven. There were 17 with *Loa loa* infection, 3 with *W. bancrofti* infection and 1 with *O. volvulus* infection. The filarial-uninfected donor cells were obtained from healthy volunteers under a protocol approved by the Institutional Review Board (IRB) of the Department of Transfusion Medicine, Clinical Center, National Institutes of Health (IRB# 99-CC-0168). Written informed consent was obtained from all subjects.

### Cell Processing

PBMCs previously cryopreserved from whole blood or buffy coats from both infected individuals and normal donors were thawed, placed over Ficoll/diatrizoate (LSM, MP Biomedicals, Santa Ana, CA), washed twice with serum-free RPMI (Gibco® Life Technologies, Grand Island, NY) supplemented with penicillin/streptomycin (Invitrogen Life Technologies, Grand Island, NY), and resuspended in RPMI supplemented with L-glutamine (Invitrogen), penicillin/streptomycin, and 10% fetal bovine serum (Gemini Bioproducts, West Sacramento, CA) for culture.

### Cell Sorting

PBMCs isolated from healthy, uninfected blood bank donors were lineage depleted using the Human Lineage Cell Depletion Kit and an Automacs cell separator (Miltenyi, Auburn, CA). The negative fraction from the separation was stained with a lineage depletion cocktail in FITC (CD2, CD3, CD14, CD16, CD19, CD56, CD235a) (eBioscience, San Diego, CA) and with anti-CD45-Pacific Blue (eBioscience), anti-cKit-Phyocoerythrin (PE) (eBioscience) and anti-IL-7Rα-Allophycocyanin (APC) (eBioscience). For the purpose of sorting, ILCs were defined as Lin−/CD45+/cKit+/IL-7Rα+ using a FACS Aria cell sorter (BD Biosciences). These cKit+ ILCs contain the ILC2 and ILC3 subsets. Gates were drawn based on back gating of the populations. The geometric mean of the percentage of ILCs in the starting population was 0.13% (range 0.03%–0.7%). The purity of the sorted cells were always greater than 87%.

### Cell culture

Sorted ILCs (∼10,000/well) were cultured in RPMI 1640 (Life Technologies) supplemented with penicillin/streptomycin, L-glutamine, and 1% human AB serum (Gemini Bio-Products, West Sacramento, CA) with or without a cytokine cocktail containing IL-25 (Peprotech, Rocky Hill, NJ), IL-33 (Peprotech), IL-7 (Peprotech) (each at 5 mg/ml) and 10U/ml rhuIL-2 (Genentech, San Francisco, CA). Supernatants were collected every 24 hours and fresh media and cytokines were added for up to 5 days. Production of the cytokines IFN-γ, IL-4, IL-5, IL-13, IL-10, and IL-17A by ILCs was determined using the Milliplex Max human cytokine panel suspension array technology assays (Luminex Millipore, Billerica, MA).

Additional sorted ILCs (2,000–5,000/well) were cultured in RPMI 1640 supplemented with penicillin/streptomycin, L-glutamine and 2% human AB serum in either media alone or in a TLR ligand cocktail containing flagellin (TLR5 ligand; Invivogen, San Diego, CA) at 0.5 ug/ml, FSL1 (TLR6 ligand; Invivogen) at 0.5 ug/ml, imiquimod (TLR7 ligand; Invivogen) at 0.1 ug/ml and the CPGs K3 and D35 (TLR9 ligand; a gift from the laboratory of D. Verthelyi) at 0.03 uM, anti-CD3 (Harlan, Indianapolis, ID) at 10 ug/ml or isotype control (BD Pharmingen) at 10 ug/ml. Supernatants were collected after 72 hours and production of eotaxin, GM-CSF, IL-3, IL-4, IL-5, IL-10, IL-1β, IL-1α, TNF-α, and IL-12p40 were measured with the Milliplex Max human cytokine panel suspension array technology assays.

Processed PBMCs from filarial-infected patients and normal uninfected controls were cultured unstimulated at 1×10^6^ cells/ml in 24 well plates, with separate wells for ILC quantitation and T-cell quantitation. Brefeldin A/Monensin were added at a concentration of 10 µl/10^6^ cells at the beginning of culture. Cells were incubated for 6 hours at 37°C. After incubation, cells were harvested and either fixed (ILC quantitation) or resuspended in FACS buffer (PBS/1% BSA/0.1% NaN_3_) (T cell quantitation).

### Flow Cytometry

Harvested and processed PBMCs were block and stained as follows. For the ILC panel, cells were stained with lineage markers (anti-CD3, anti-CD4, anti-CD8, anti-CD16, anti-CD14, anti-CD19 [eBioscience], anti-CD56, anti-FcεR1, anti-CD11b and anti-CD11c [Biolegend, San Diego, CA]), all labeled with Pacific Blue, anti-CD45-Pacific Orange (Invitrogen), anti-cKit-PerCP-eF710 (eBioscience) and anti-IL-7Rα-APC-Cy7 (eBioscience). Cells were then permeabilized and stained with anti-human IL-13-FITC (eBioscience) and data were collected on a BD FACS Canto II (BD Biosciences).

For the T cell panel, PBMCs were stained with anti-CD3-V500, anti-CD4-Qdot 605 and anti-CD8-PE-Texas Red. Cells were then fixed, permeabilized and stained with anti-IL-4-FITC, anti-IL-2-PerCP, anti-IL-10-Pacific Blue, anti-IL-22-APC, anti-TNF-α, anti-IL-17A-APC-Cy7, anti-IL-5-PE and anti-IFN-γ-PE-Cy7 for 30 minutes. Data were collected on a BD LSRFortessa (BD Biosciences). All flow data were analyzed using FlowJo version 9.4.10.

### RNASeq

RNA was isolated from sorted ILC (n = 6 donors) and lineage depleted PBMCs (control group, n = 2 donors) using standard methods as described previously [12]. The isolated RNA was purified using RNAeasy (Qiagen, Valencia, CA). The concentration and purity of the RNA was determined using an Agilent Bioanalyzer 2100 (Agilent, Santa Clara, CA). Poly-A selected mRNA libraries were constructed from 1 µg total RNA using the Illumina TruSeq RNA Sample Prep V2 Kits (Illumina, San Diego, CA) according to the manufacturer’s instructions except where noted. The cDNAs were fragmented to ∼275 bp using a Covaris E210 (Covaris, Woburn, MA). Amplification was performed using 10 cycles to minimize the risk of over-amplification. Unique barcode adapters were applied to each library and equal volumes of individual libraries were pooled and run on a MiSeq (Illumina). The libraries were then re-pooled based on the MiSeq demultiplexing results. Pooled libraries were sequenced on two lanes of a HiSeq2000 (Illumina) using version 3 chemistry. The data were processed using RTA version 1.13.48 and CASAVA 1.8.2. The raw data were deposited at the Sequence Read Archive (SRA) of the National Center for Biotechnology Information (NCBI) under the following identities: Bioproject PRJNA210757, Biosample SRX319050 and runs SRR927420, SRR927421, SRR927423, SRR927424, SRR927428 and SRR927431 for patients and runs SRR927436 and SRR927437 for control reads. All reads were mapped to available human genome transcripts (NCBI Refseq database dated 12/21/2012) using in house software developed by J. Ribeiro.

### RT-PCR

RNA was extracted from previously cryopreserved PBMCs from a normal donor and cryopreserved sorted cKit+ ILCs using standard methods as previously described [12]. cDNA generation was performed using random hexamers and reverse transcriptase (Life Technologies). Expression of *EPS8* (Assay ID# Hs00610286_m1), *FARP1* (Assay ID# HS00195010_m1), *KIT* (Assay ID# Hs00174029_m1), and *XCL1* (Assay ID# Hs00751481_s1) was determined by quantitative real-time PCR using pre-developed Taqman ™ reagents (Life Technologies) on an ABI 7900HT. The threshold cycle (CT) was calculated for the 18S control and the genes of interest and used to determine the relative transcript levels. The formula 1/ΔCT, was used to compare the relative transcript levels, where ΔCT is the difference between the CT of the target gene and the CT of the corresponding endogeneous 18S reference.

### Statistical Analyses

Unless noted otherwise, geometric means were used as measures of central tendency. Nonparametric statistics were used throughout; the Mann-Whitney test was used for comparisons of frequencies and the Spearman Rank Correlation analysis was used for all correlation analyses. Statistical analyses were performed in GraphPad Prism v6.

For the RNAseq data, paired normalized fold changes between libraries were calculated by combining the number of reads for a given gene across all ILC and all control samples and then dividing all ILC by all controls +1. Normalization of the reads was obtained by multiplying the numerator by the ratio of the total number of reads to all genes from each paired comparison. X^2^ tests were used to detect significant differences between samples when the minimum expected value was larger than 5 and P<0.05. A 2-fold change (up or down) was considered of interest when also statistically significant. To further improve the stringency a cutoff of p<0.05 from a Student’s t-test of cKit+ ILCs versus controls was used. The resulting data were mapped to a hyperlinked Excel spreadsheet that also contained blast comparisons of the RNA data to several databases to facilitate data annotation. An automatic functional classification of the transcripts was performed using a program written by J. Ribeiro, based on a ∼250 word vocabulary targeting matches to Swissprot, Gene Ontology, CDD and RefSeq vertebrate databases. This Excel file is available at http://exon.niaid.nih.gov/transcriptome/H_sapiens_nuocytes/Nuocytes.xlsx. Significantly differentially regulated genes were analyzed using Ingenuity 14855783 and JMP v10.

## Results

### cKit+ ILCs are increased in filarial infected patients

**Figure 1:**
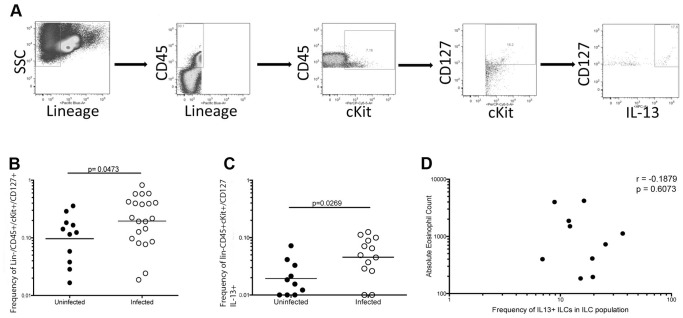
cKit+ ILCs are increased in filarial-infected individuals. (A) Representative flow diagram from a filarial-infected individual demonstrating the cKit+ ILC gating strategy. Lin−/CD45+/cKit+/CD127+ cells were identified as cKit+ ILCs of which 28% were positive for IL-13 production. (B) Frequency of cKit+ ILCs (Lin−/CD45+/cKit/CD127+ cells) in filarial-uninfected (filled circles, n = 11) and filarial infected (open circles, n = 21). Horizontal bars are the geometric mean (GM) of the frequencies. (C) Frequency of IL-13+ cKit+ ILCs (Lin−/CD45+/cKit+CD127+/IL-13+ cells) in filarial-uninfected (filled circles, n = 11) and filarial-infected (open circles, n = 21) individuals. Horizontal bars are the geometric mean (GM) of the frequencies. (D) Correlation between the absolute eosinophil count and the frequency of IL-13+ cKit+ ILCs in the patients and uninfected individuals. The p value and r value from the Spearman rank correlation are shown.

ILCs have been associated with the immunity to helminth parasites in rodents (1–4). Thus, we developed a strategy to identify these cells in human peripheral blood using the definition of ILCs as Lin−/CD127+ cells (flow strategy not shown). We also examined cKit+ ILCs using the flow strategies outlined in Figure 1A and Figure S1. We then examined the frequency of total ILCs and cKit+ ILCs in the peripheral blood of filarial-infected (Fil+) patients and in healthy, uninfected (NL) individuals.

While there was a modest increase in the frequency of total ILCs (defined as Lin−/CD127+) (data not shown) in Fil+ individuals, there was a statistically significant ∼2-fold increase (p = 0.0473) in the frequency of the cKit+ ILCs in the Fil+ (geometric mean [GM] frequency = 0.195 (range 0.0037–0.857) (Figure 1B) compared to the frequencies found in NL (GM = 0.096; range 0.0029–0.0369). Moreover because this cKit+ ILC population is largely comprised of ILC2 and ILC3 subsets and because filarial infection has been associated with increases in CD4+ Th2-like cells, we assessed the frequencies of IL13+cKit+ILCs in all donors (Figure 1C and Figure 1A for a representative flow diagram). As can be seen, the frequency of IL-13 producing cKit+ ILCs in the Fil+ population was ∼ 2- to 3-fold higher (GM = 0.046, range 0.001–0.125) than seen in the NL (GM = 0.019, range 0.001–.072) group. Unlike data recently described in the mouse [13]the frequency of ILC2s in the blood did not correlate with the absolute eosinophil counts (p = 0.9949, r = 0.0011232) (Figure 1D).

**Figure 2:**
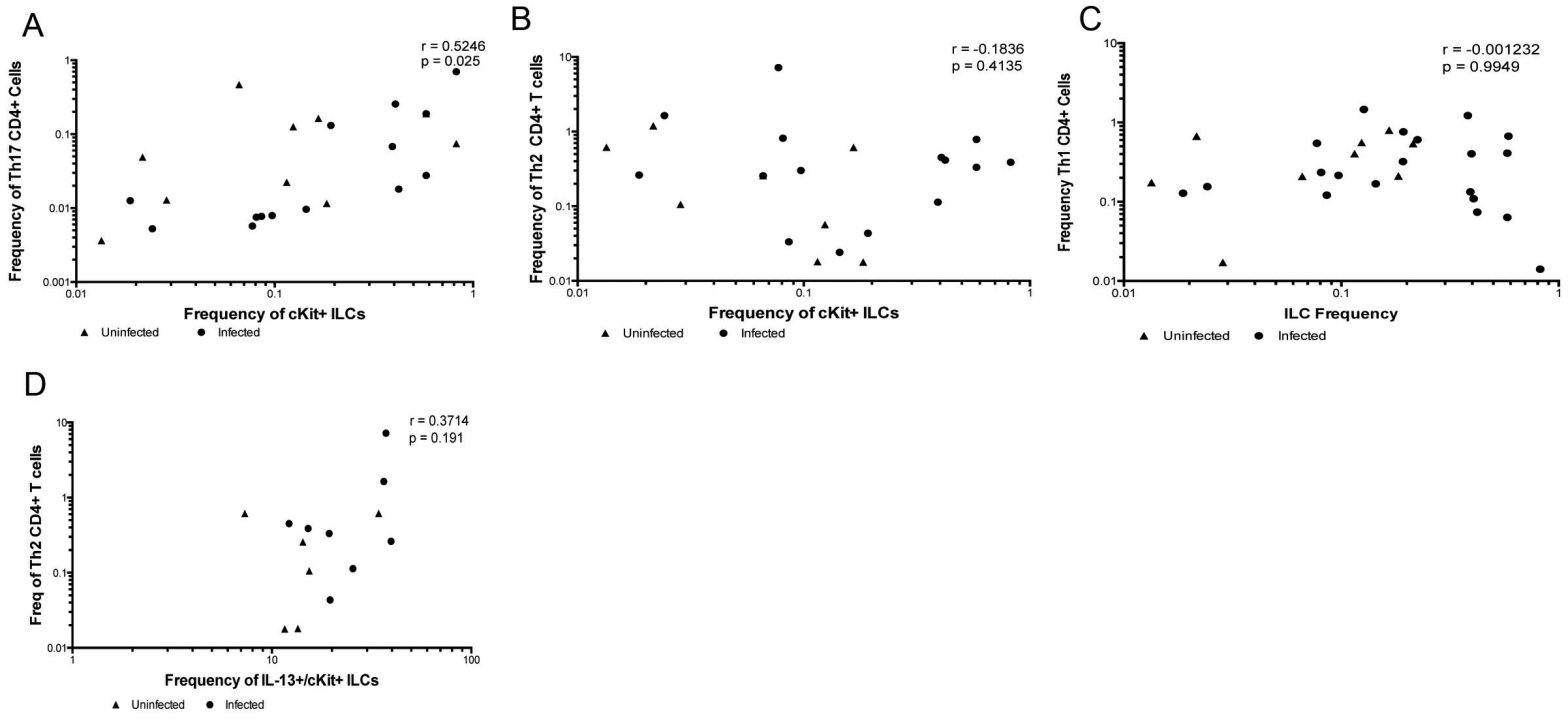
The frequency of cKit+ ILCs in peripheral blood correlates with the frequency of Th17 cells. Correlation of the frequencies of cKit+ ILCs and the frequencies of Th17 (A), Th2 (B), and Th1 (C) CD4+ T cells in 21 filarial-infected (circles) and 11 filarial-uninfected individuals (triangles). The p and r-values from the Spearman rank correlation analysis are shown. (D) Correlation of the frequencies of IL-13 producing cKit+ ILCs and Th2 CD4+ T cells from 8 filarial-infected and 6 uninfected individuals. The p-value and r-value from the Spearman rank correlation analysis are shown.

To assess the relationship between the frequencies of cKit+ ILCs and the frequencies of Th subsets in CD4+ T cells producing Th1 (IFN-γ, TNF-α)-, Th2 (IL-4, IL-5, IL-10)- and Th-17 (IL-22 or IL-17)-associated cytokines, frequencies of all of these populations were assessed concurrently from the same individual. As can be seen (Figure 2) the frequency of cKit+ ILCs correlated strongly with the frequency of CD4+ T cells producing IL-17A (r = 0.5246, p = 0.0267) (Figure 2A). However, the frequencies of the these cKit+ ILCs failed to correlate with the frequencies of Th2 cells – that is CD4+ T cells producing IL-4 and/or IL-5 (r = −0.2215, p = 0.287; Figure 2B), with the frequencies of Th1 cells (CD4+ T cells producing IFN-γ; r = −0.001, p = 0.995; Figure 2C), or with those producing any of the other cytokines (IL-10, IL-22) examined (data not shown). In addition, the frequencies of ILCs producing IL-13 (ILC2) did not correlate with the frequencies of Th2 cells either (Figure 2D).

### Purified cKit+ ILCs produce Th2-, Th17- and Th1-related cytokines when stimulated

**Figure 3:**
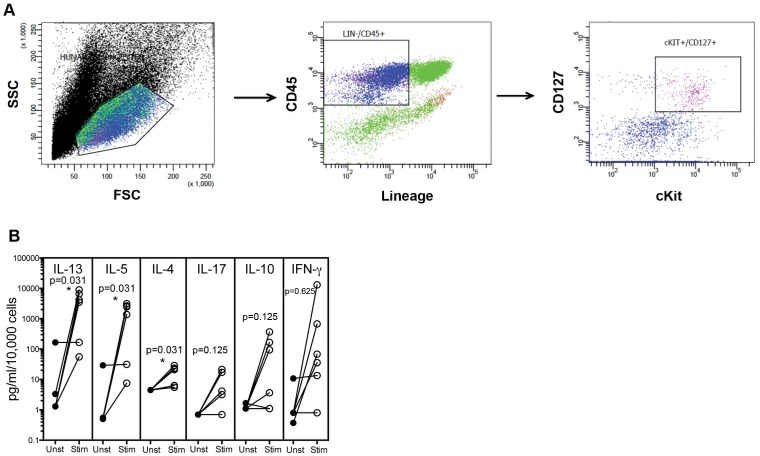
Cell sorting yields a purified ILCs that produces Th2-, Th17- and Th1-related cytokines. (A) Representative flow diagram of the strategy used for cell sorting of cKit+ ILCs (B) Sorted cKit+ ILCs (n = 5) cultured with media alone (closed circles-Unst) or with a cytokine cocktail consisting of rhIL-2, IL-7, IL-25 and IL-33 (open circles-Stim) for 3 days.

Given that the cKit+ ILCs were increased in the Fil+ population, we set out to examine these cells in uninfected individuals. Using the definition of Lin−/CD45+/cKit+/CD127+, ILCs were sorted from normal human peripheral blood by flow cytometer-based cell sorting to yield relatively pure ILC populations (Figure 3A). On average, the cKit+ ILCs represented ∼0.2% of the lineage-depleted PBMCs when sorted. When these sorted ILCs were stimulated with rhIL-2, IL-7, IL-25 and IL-33, there was a statistically significant GM 1382-fold increase in IL-13 (p = 0.0313), a 1442-fold increase in IL-5 (p = 0.0313) and a 4-fold increase in IL-4 (p = 0.0313) production by cytokine-stimulated ILCs (Figure 3B) compared to cytokine-unstimulated cKit+ ILCs. For the ILC2 population, we could estimate that ILC2s produced ∼43 fg of IL-5/cell, 2 fg of IL-4/cell and 140 fg of IL-13/cell levels that, for IL-5 and IL-13 at least, greatly exceed the per cell production of these cytokines by CD4+ Th2 cells. In addition, there was a 5.21-fold rise in IL-17A, a 14.56-fold increase in IL-10, and a 72.16 fold increase in IFN-γ, which supports previous data that both ILC2s and ILC3s can express cKit [5].

To verify the purity of the sorted cKit+ ILCs and to ensure that there were no contaminating CD3+ cells, additional sorted cKit+ ILCs were exposed to anti-CD3 or an isotype control antibody, and the level of cytokine production assessed. Unlike the ILC-specific stimulation shown in Figure 3, stimulation with anti-CD3 failed to induce any measurable production of IL-4, IL-5, IL-10 or IFN-γ (data not shown). These data suggest that the purified cKit+ ILCs were free from any contaminating T cells.

### cKit+ ILCs have a unique transcription profile

**Figure 4:**
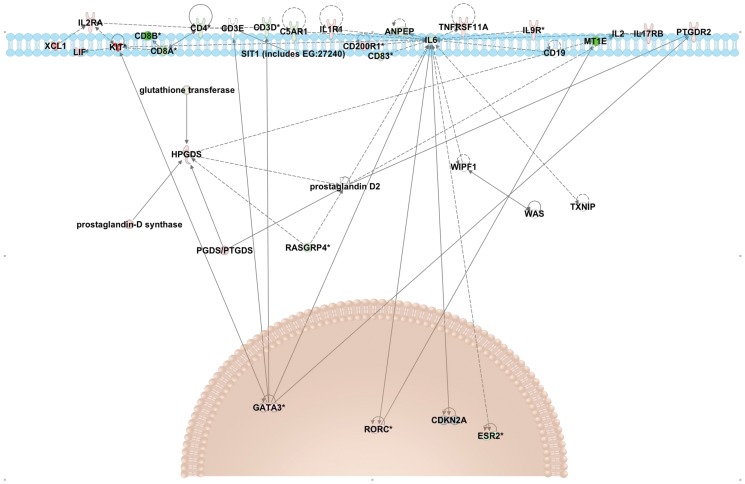
At homeostasis cKit+ ILCs express transcripts associated with the ILC2 and ILC3 subsets. Transcripts differentially expressed between ILCs and lineage depleted PBMCs (control cells). The upregulated and downregulated transcripts from the RNASeq analysis were put into Ingenuity Pathway Analysis for graphical illustration. Pathway depicts genes that are upregulated (red) and downregulated (green) in cKit+ILCs in comparison to lineage-depleted PBMCs and their presumed location in the cell. The top of the figure depicts the cell membrane, with the nucleus towards the bottom of the figure.

RNA was extracted from sorted cKit+ ILCs (n = 6) and from the lineage depleted peripheral blood mononuclear cells (PBMCs) from which the cKit+ ILCs were sorted (n = 2) and expression profiles of each of these cell populations were assessed by RNAseq. Our sorted Lin−/CD45+/cKit+/CD127+ population had an increase in the expression of GATA3 and RORC (which encodes RORγt), transcription factors associated with ILC2 and ILC3 subsets respectively [5] (Figure 4). Transcripts for PTGDR2, which encodes CRTH2, and IL-17RB, were also increased in cKit+ ILCs compared to controls (Figure 4). These data confirm that by selecting cKit+ ILCs, we are capturing ILC2s and ILC3s (Figure 4).

**Figure 5:**
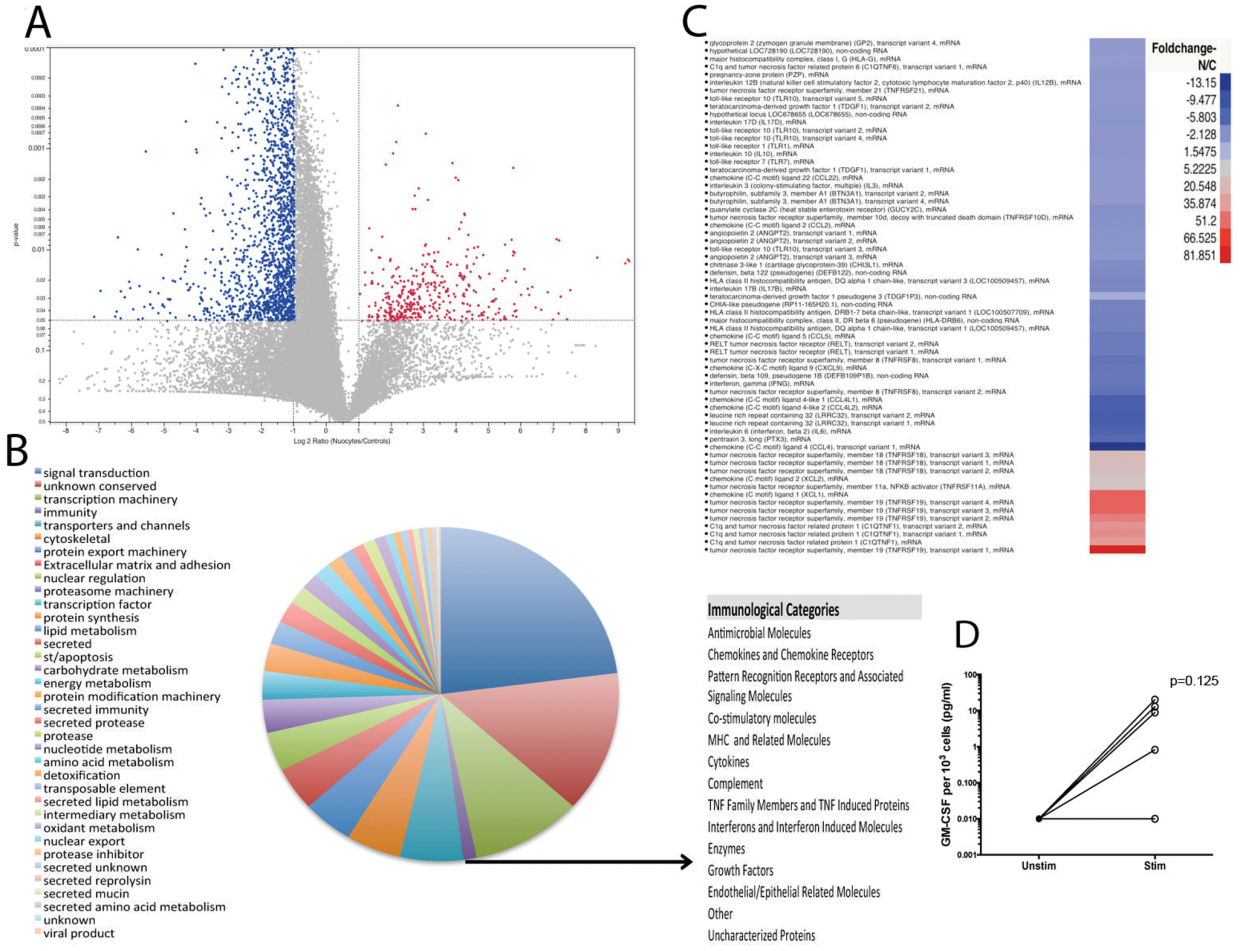
The majority of expressed transcripts in cKit+ ILCs are for signal transduction and transcription machinery. (A) Volcano plot depicting all transcripts with those upregulated shown in red (n = 472) and those downregulated shown in blue (n = 2371). (B) Pie chart showing the functional classification of all transcripts identified. Sub-categories of the immunologically relevant genes are listed (B) with a full listing of all the genes classified as immunologically relevant in Table S1. (C) A heat map of the immunologically relevant transcripts of cKit+ ILCs (n = 6) compared to lineage depleted PBMCs (n = 2). (D) Sorted cKit+ ILCs (n = 6) cultured with media alone (closed circles – Unst) or with a cocktail of TLR ligands for TLR5, TLR6, TLR7 and TLR9 (open circles – Stim) for 3 days.

All of the transcripts that mapped to the human transcriptome are represented as a volcano plot (Figure 5A) and listed in their entirety at http://exon.niaid.nih.gov/transcriptome/H_sapiens_nuocytes/Nuocytes.xlsx. Of these 42,434 transcripts, 472 transcripts were found to be statistically significantly upregulated (or cKit+ ILC-biased) in the cKit+ ILC population (Figure 5A). In contrast there were 2371 downregulated (Lin-PBMC biased) transcripts in the ILCs (upper left quadrant) based on the same criteria. When all of the 42,434 ILC-expressed genes that mapped to the human transcriptome were classified by function (Figure 5B), those associated with signal transduction (n = 8652) and transcription machinery (n = 3792), as well as those without known function (n = 5157) were the most abundant. Further evaluation of immunologically relevant transcripts (n = 645) identified those associated with cytokines, chemokines, chemokine receptors, TLRs, and MHC molecules, among others (Figure 5B). The full list of sub-classified immunologically relevant genes is located in Table S1. As can be seen in Figure 5C, the immunologically relevant transcripts that showed significantly increased expression in cKit+ ILCs include tumor necrosis family receptor superfamily member 19 (TNFRFS19) (81-fold), C1q related protein (51.2-fold) and chemokine ligand 1 (66-fold). These data suggest that these ILCs are equipped to sense and respond to pathogens.

Among the TLRs expressed by cKit+ ILCs, TLR5, TLR6, TLR7 and TLR9 were the most abundant. Since stimulation of ILCs has primarily been described using barrier-associated cytokines such as IL-33 and IL-25, we sought to determine if ILCs could respond to signals independent of these cytokines. Sorted cKit+ ILCs were cultured with or without a TLR ligand cocktail for TLR5, TLR6, TLR7 and TLR9, as well as with the ligands for each of these TLRs separately. The purified cKit+ ILCs responded to both the combination of TLR ligands and specifically to the TLR6 ligand by producing an increased amount of GM-CSF as compared to the media alone (127.6-fold) (Figure 5D), although TLR-signaling in the cKit+ ILCs failed to induce the more prototypic cytokines (e.g. IL-5, IL-17A, IFN-γ). Nevertheless, the response of cKit+ ILCs to TLR stimulation suggests that this cell type can respond directly to pathogens and not only to cytokine signals sent by accessory cells.

Table 1 contains a list of the top 50 most upregulated genes in the cKit+ ILCs grouped by functional classification. EPSL8, KIT, FARP1, and XCL1 were confirmed to be upregulated in cKit+ ILCs by RT-PCR (Figure S2). The single most upregulated gene in the cKit+ ILCs compared to the control cells is an epidermal growth factor substrate 8 (EPS8)-like molecule (Table 1), a molecule that functions in intracellular cytoskeletal organization through actin refolding [14]–[16]. Taken from the relative abundance of EPS8 and the other highly upregulated transcripts is the suggestion that the cKit+ ILC population identified in the blood has an increased proliferative capacity compared to other lineage negative cells in the blood.

**Table 1:** Top 50 upregulated transcripts in ILCs.

Sequence Name	Expression in ILCs	Expression in Controls	NCBI Description
gi_320118888	649.57	0.00	EPS8-like 3 (EPS8L3), transcript variant 2
gi_320118887	631.85	0.00	EPS8-like 3 (EPS8L3), transcript variant 1
gi_320118889	596.32	0.00	EPS8-like 3 (EPS8L3), transcript variant 3
gi_63054849	327.84	0.00	deoxynucleotidyltransferase, terminal (DNTT), variant 1
gi_63054851	327.51	0.00	deoxynucleotidyltransferase, terminal (DNTT), variant 2
gi_156415982	184.43	0.01	transmembrane protease, serine 11E (TMPRSS11E)
gi_159032536	171.52	0.00	FERM, RhoGEF (ARHGEF) 1 (FARP1), variant 2
gi_227430282	150.13	0.00	hepsin (HPN), transcript variant 2
gi_194239680	145.31	0.01	tubulin tyrosine ligase-like member 10 (TTLL10) variant 1
gi_149944492	143.48	0.00	dynein, axonemal, heavy chain 14 (DNAH14), variant 3
gi_194239677	137.67	0.01	tubulin tyrosine ligase-like member 10 (TTLL10), variant 2
gi_194578882	127.83	0.00	nuclear factor of activated T-cells, 4 (NFATC4), variant 2
gi_148005038	113.88	0.01	v-kit Hardy-Zuckerman 4 (KIT) variant 2
gi_148005048	113.87	0.01	v-kit Hardy-Zuckerman 4 n(KIT), variant 1
gi_254939711	99.07	0.01	collagen, type XI, alpha 2 (COL11A2), variant 4
gi_149588714	87.92	0.00	V-set and transmembrane domain containing 2 like (VSTM2L)
gi_325120956	81.85	0.01	TNF receptor superfamily, member 19 (TNFRSF19), variant 1
gi_227430281	76.59	0.01	hepsin (HPN), transcript variant 1
gi_291167770	74.30	0.01	solute carrier family 22, member 20 (SLC22A20), variant 1
gi_63252890	69.04	0.01	prolyl 4-hydroxylase, alpha polypeptide II (P4HA2), variant 2
gi_63252892	64.46	0.01	prolyl 4-hydroxylase, alpha polypeptide II (P4HA2), variant 3
gi_194595470	58.25	0.00	guanylate cyclase 1, soluble, alpha 3 (GUCY1A3), variant 1
gi_116295253	58.24	0.02	heparan sulfate 6-O-sulfotransferase 2 (HS6ST2), variant S
gi_149408143	57.62	0.02	TOX high mobility group box family member 2 (TOX2), variant 1
gi_75709190	56.20	0.02	cytochrome P450, family 2, subfamily E, polypeptide 1 (CYP2E1)
gi_116295255	56.19	0.02	heparan sulfate 6-O-sulfotransferase 2 (HS6ST2), transcript variant
gi_120587024	55.72	0.02	SH3 and multiple ankyrin repeat domains 1 (SHANK1), mRNA
gi_325120960	54.65	0.02	TNF receptor superfamily, member 19 (TNFRSF19), variant 4
gi_312434026	54.63	0.02	chemokine (C motif) ligand 1 (XCL1), mRNA
gi_325120958	54.42	0.02	TNF receptor superfamily, member 19 (TNFRSF19), variant 3
gi_93141213	54.17	0.02	sodium channel, voltage-gated, type II, alpha (SCN2A), variant 3
gi_194595481	52.68	0.01	guanylate cyclase 1, soluble, alpha 3 (GUCY1A3), variant 7
gi_4507246	48.08	0.02	SH3 and cysteine rich domain (STAC), mRNA
gi_81295799	46.66	0.01	paired box 8 (PAX8), transcript variant PAX8A, mRNA
gi_334724446	45.85	0.01	homeobox A6 (HOXA6), mRNA
gi_325120957	45.79	0.02	TNF receptor superfamily, member 19 (TNFRSF19), variant 2
gi_93141211	45.26	0.02	sodium channel, voltage-gated, type II, alpha (SCN2A), variant 2
gi_269973870	44.77	0.00	kelch-like 13 (Drosophila) (KLHL13), transcript variant 6, mRNA
gi_217272860	43.15	0.00	prolyl 4-hydroxylase, alpha polypeptide II (P4HA2), variant 4
gi_38372915	41.42	0.02	C1q and tumor necrosis factor related protein 1 (C1QTNF1), variant 1
gi_115392132	40.59	0.02	collagen, type XXIV, alpha 1 (COL24A1), mRNA
gi_156071535	39.91	0.02	C1q and tumor necrosis factor related protein 1 (C1QTNF1), variant 2
gi_48928036	37.62	0.03	FERM, RhoGEF and pleckstrin domain protein 1 (FARP1), variant 1
gi_38372912	37.54	0.02	C1q and tumor necrosis factor related protein 1 (C1QTNF1), mRNA
gi_310120751	36.14	0.00	hypothetical LOC100506259 (LOC100506259), miscRNA
gi|194595471	35.87	0.01	guanylate cyclase 1, soluble, alpha 3 (GUCY1A3), variant 2
gi|149408142	36.02	0.03	TOX high mobility group box family member 2 (TOX2), variant 3
gi|149408140	35.46	0.03	TOX high mobility group box family member 2 (TOX2), variant 4
gi|119395749	36.06	0.03	keratin 1 (KRT1), mRNA

**Figure 6:**
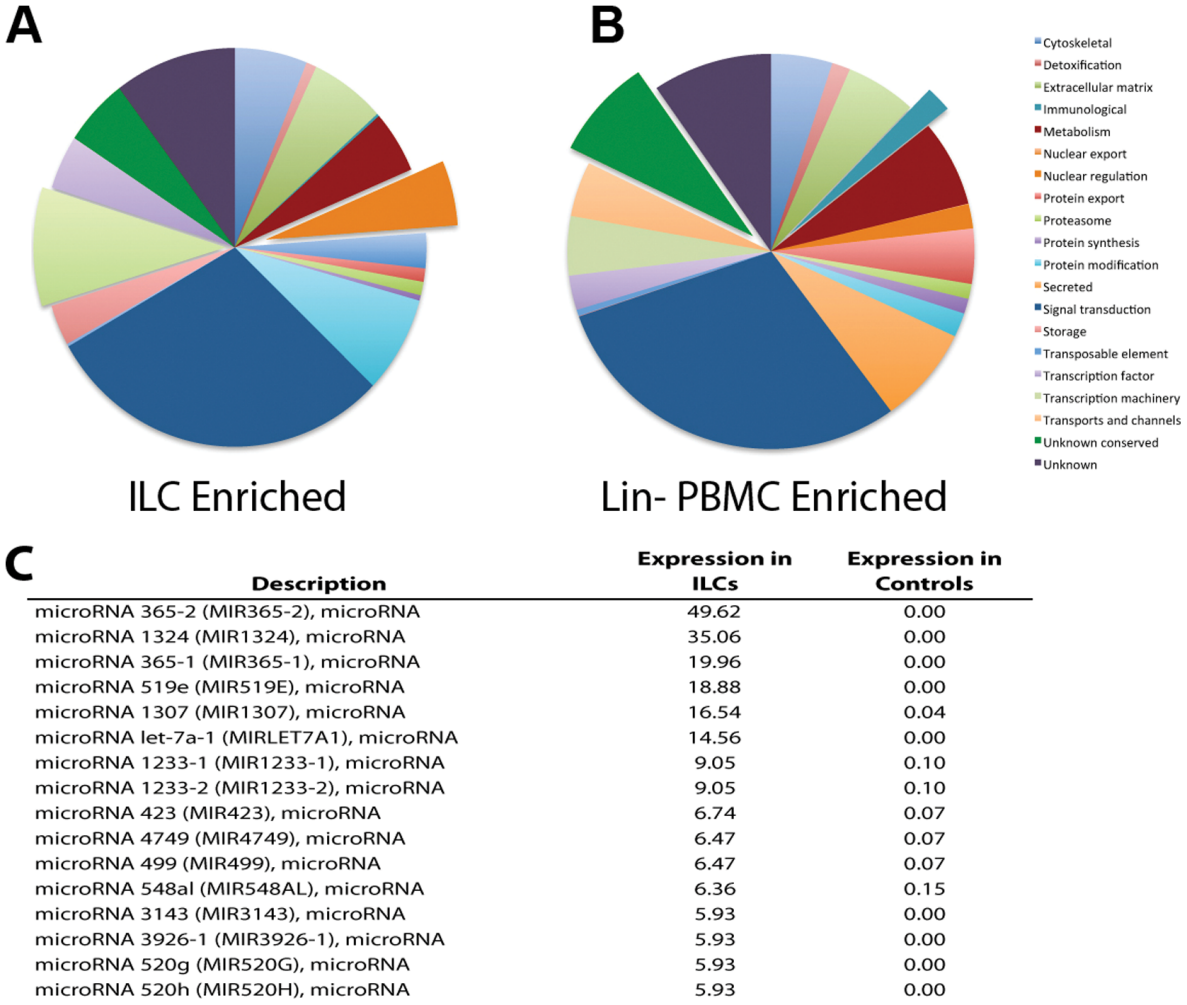
cKit+ ILCs express more genes for nuclear regulation and transcription machinery and a unique microRNA signature. (A) Pie chart depicting a functional analysis of the transcripts enriched in ILCs. Pulled out slices are functional categories significantly increased in ILCs compared to lineage depleted PBMCs. (B) Pie chart showing a functional analysis of transcripts enriched in lineage depleted PBMCs compared to ILCs. Pulled out slices are categories significantly increased in lineage depleted PBMCs. (C) A list of the 10 most abundantly expressed microRNA precursors in ILCs and their expression levels in controls (lineage depleted PBMCs).

Grouping the cKit+ ILC biased genes by function identified signal transduction genes as the most prominent (n = 142; Figure 6A). One sub-category in this group represented cytokine receptors, specifically the IL-17B and IL-17E receptors as well as the IL-18R, IL-1R and IL-9R (data not shown). Another sub-category in the signal transduction functional class contained growth factors and their receptors. In addition to promoting growth in various cells, growth factors can mediate functions such as migration, adhesion and endocytosis [17], [18]. In sum, many of the transcripts associated with signal transduction were related to conveyance of extracellular signals, suggesting cKit+ ILCs are capable of surveying their environment and responding quickly to external stimuli. Within the upregulated functional categories, comparison of cKit+ ILCs with control cells demonstrated that cKit+ ILCs expended significantly more cellular energy to transcription machinery (p = 0.002) and nuclear regulation (p = 0.002) than did the Lin- controls and expressed significantly fewer transcripts in the categories of immune process (p = 0.0026; Figures 6A and B).

Among the downregulated genes involved broadly in signal transduction (n = 688) (Figure 5B), those pro-apoptotic transcripts (e.g. caspase recruitment domains 12 and 6 as well as a BCL2-like transcript and caspase 12) were diminished in the cKit+ILCs as compared to the controls (n = 38 and n = 7 respectively). Of particular interest in the context of the immune response, the chemokines CCL2, CCL3, CCL4, CCL5, and CXCL9 were present in the controls and were not present in the cKit+ ILCs (data not shown), a finding that suggests that these cKit+ ILCs, play a specific role in the innate immune response.

A number of microRNA precursors were also identified in this RNAseq analysis. Among the 1374 microRNAs (mirs) identified in this analysis in either (or both) cell populations, 16 mirs had >6-fold higher expression in the cKit+ ILCs while expression was absent or very low in controls (Figure 6C). Mirs 365-2 and 365-1 (49.6 and 20, respectively), as well as mir-1324 (35.1) and mir-519e [19] were very highly expressed in cKit+ ILCs and not by control cells (Figure 6C).

## Discussion

Since innate immune cells produce the cytokines responsible for Th2 and Th17 differentiation and had not yet been investigated in the context of human parasitic infection, we identified ILCs, and a specific subset of cKit+ ILCs, in the peripheral blood and attempted to assess their role in human filarial infection. We found that filarial-infected subjects had an ∼2-fold expansion of cKit+ ILCs compared to those who were filarial-uninfected as well as a significant increase in cKit+ ILCs producing IL-13 (ILC2s). Because the microfilariae (the circulating larval stage of the filarial parasites) have been shown to modulate the function of other circulating innate cells such as dendritic cells (DCs) [20] and monocytes [21], it is likely that exposure to microfilariae of peripheral ILCs (or their precursors) leads either to the activation of these cells directly or indirectly through the induction of cytokines from endothelial (or other barrier associated) cells that in turn leads to the increased frequencies seen in filarial-infected individuals.

We were able to demonstrate a strong correlation between the frequency of circulating cKit+ ILCs and the frequency of CD4+ T cells producing IL-17 (Th17 cells) but not for Th2 or Th1 cells (Figure 2). A recent study has identified inflammatory DCs as a potential source of the cytokines responsible for driving Th17 differentiation [22]; however, there is no consensus as to how the Th17 response is initiated and maintained. Thus, cKit+ ILCs may play a role in the initiation and or maintenance of the Th17 response. The lack of correlation between the frequency of cKit+ ILCs and the frequency of Th2 cells or the frequency of ILC2s (as defined by IL-13 production) may be due to the surface markers used to distinguish ILCs in this and other studies. While all previously identified ILC subsets are lineage- and CD127+, those that are also CD117+ are most likely LTi-like (ILC3 subset distinguished by IL-17 and IL-22 production) despite the fact that a proportion of ILC2s also express CD117 [5]. The surface markers associated with ILC2s are not completely delineated. In humans, blood and nasal tissue ILCs express CRTH2 [6]; however, other studies have shown that CRTH2 is not a marker of ILC2s in the skin [8]. Thus, the surface molecules associated with the ILC2 population in humans are not fully determined and there may be some overlap of cytokine expression from the different subsets.

A recent report found that in mice, long-lasting ILC2s in the tissue are responsible for maintaining eosinophil numbers through the constitutive expression of IL-5 and regulate eosinophil accumulation through IL-13 production activated during type 2 inflammation [13]. While the high levels of IL-5 and IL-13 secretion by the human peripheral blood cKit+ ILCs in this study suggest that human ILC2s may play a parallel role with regard to eosinophil maintenance and recruitment, there was no correlation between the frequency of ILC2s in the peripheral blood and the absolute eosinophil numbers (data not shown). The lack of a correlation does not preclude a relationship between ILC2s and eosinophils in humans; however, an examination of tissue-resident eosinophils and ILCs would be needed to fully investigate the relationship between these two cell types in humans.

To date the primary function assigned to ILCs (including the cKit+ subsets) has been that of cytokine production, although there has been recent evidence of direct interaction of ILCs with T cells [23] and mast cells [8]. To gain a better understanding of the function and regulation of the cKit+ ILCs, PBMCs from normal blood donors were lineage depleted and flow cytometer-sorted for the ILC population and assessment of cytokine production (Figure 3B). Our data confirm the marked cytokine production of ILCs and demonstrate that even though these cKit+ ILCs represent a rare population in the peripheral blood (0.01%–0.1%), they have the capacity to produce large amounts of cytokines that would influence the microenvironment surrounding innate and adaptive immune cells.

Although the consensus is that ILCs are not stem or progenitor cells [5], our data show increased expression of genes linked to proliferation (EPS8 and DNTT) and to differentiated ILCs (Gata3 and Rorγt), findings that substantiate the concept that the cKit+ ILCs in the blood retain an increased proliferative capacity but have already differentiated. Moreover, as shown by RNASeq analyses, control cells (non-ILCs) expressed many more apoptosis-related transcripts than did the purified cKit+ ILCs. This apparent downregulation of pro-apoptotic pathways in cKit+ ILCs may be one mechanism to explain the enhanced survival of the ILC2s seen in the mouse [13].

cKit+ ILCs express the full range of TLRs (1–10) suggesting that they sense pathogens without signals from epithelial or endothelial cells (Table S1). Indeed, when purified cKit+ ILCs were stimulated with TLR ligands, they produced increased amounts of GM-CSF compared with those cells in media alone (Figure 5D). Indeed, a recent investigation has shown that in mice that lack MyD88, a key mediator of TLR signaling, Th2 responses are significantly diminished, suggesting that the TLR signaling pathway may contribute to ILC-mediated Th2 responses [24]. Additionally, MHC Class-I and MHC Class-II transcripts were identified in cKit+ ILCs, signifying that not only do these ILCs have the potential to directly sense pathogens, but they may also be capable of internalizing and presenting antigens (Table S1). Our observations are reflective of a recent study in mice that found MHCII expressed by ILC2s localized to the skin [8].

The cKit+ ILCs also expressed an array of chemokines and chemokine receptors, many of which are likely secreted (Table S1). However, while these chemokines and receptors are expressed in cKit+ ILCs, some chemokines (CCL2, CCL5, CXCL9, and CCL4) were expressed at lower levels than seen in non-ILCs, while only chemokine ligand 1 (XCL1) was upregulated in the cKit+ ILCs. These data suggest that cKit+ ILCs may not function to attract other immune cells such as monocytes (CCL2), eosinophils or basophils (CCL5), but may preferentially attract T cells through XCL1.

Interestingly, there were a number of microRNA precursors that were highly expressed in cKit+ ILCs and not in Lin- control cells (Figure 6C). MicroRNA (mir) 365-1 and 2 were the most upregulated microRNAs and have been implicated in the negative regulation of IL-6 [16]. Mir-519 and -519E were also upregulated in cKit+ ILCs; these microRNAs have been shown to inhibit cell growth and thus promote cell survival [25]whereas mir-423 promotes cell growth by regulating the G(1)/S transition in the cell cycle in hepatocellular carcinoma cell lines [26]. The mir-520 group suppresses NFκB and TGF-β signaling, again in the context of cancerous cells [27], but given the roles of both signaling molecules in cytokine signaling and regulation, it is likely that this microRNA has alternative functions in these ILCs. Taken together, the expression profile of microRNAs suggests that the regulation by these microRNAs may be in part responsible for the differentiation of ILCs and their maturation from a precursor lymphoid cell.

At homeostasis, the cKit+ ILCs express transcripts for Rorγt and GATA3 but not for Tbet, confirming that this subset of ILCs is comprised of ILC2s and ILC3s. Genes associated with the surface receptors CRTH2 (PTGDR2), IL17RB (receptor for IL-17B and IL-25), IL-2R and IL-9R (Figure 4) were also upregulated in the cKit+ ILCs. In addition, cKit+ ILCs upregulated HPGDS, that may represent another effector molecule by which these ILCs influence Th subset differentiation. Of late, two new regulators of ILC2 development have been reported in mice [28] and humans [29]. Growth factor independent 1 transcription repressor (Gfi1) was shown to influence not only the development of ILC2s but also their response to IL-33 in mice [26]. In humans, the strength of NOTCH1 signaling, with low signals promoting T cells and strong signals promoting ILC2s, was found to be critical in the development of ILC2s [27]. Transcripts for both Gfi1 and NOTCH1 were found in the transcriptome of cKit+ ILCs in this study. Additionally, the expression of both of these transcripts was increased in the cKit+ ILCs as compared to the controls. The upregulated expression of Gfi1 and NOTCH1 in these ILCs again confirms that the cKit+ ILCs are made up of ILC2s in addition to ILC3s. However, recent data from our lab investigating ILC subsets in human whole blood, showed that neither GATA3 nor Tbet were sufficient to completely distinguish ILC1s or ILC2s (data not shown) which may indicate that ILCs at homeostasis in peripheral blood are relatively undifferentiated/promiscuous.

In summary, the fact that cKit+ ILCs are increased in the peripheral blood during human filarial infection that reflects the expanded Th17 and Th2 cell populations in these infections highlights a role for these versatile innate cells in human parasitic infection. Additionally, a full transcriptome of cKit+ ILCs at homeostasis is presented here, providing insight into the function and regulation of these cells. Specifically, the capacity of ILCs to sense and respond to external stimuli as well as their increased proliferative capacity are evident.

## Supporting Information

Figure S1 **Fluorescence minus one controls for ILC flow cytometry gating strategy.** Fluorescence minus one (FMO) controls were obtained by adding all but one antibody stain to PBMCs isolated from normal patients for (A) the Lineage panel, (B) CD45, (C) cKit and (**D**) CD127.
(TIF)

Figure S2 **Top upregulated transcripts in the cKit+ ILCs transcriptome are confirmed with RT-PCR.** RNA was extracted from cryopreserved PBMCs and sorted cKit+ ILCs from normal donors; mRNA levels were measured by real-time PCR and normalized to the levels of 18S ribosomal RNA. Results are shown as 1/ΔCT for normal PBMCs (n = 1; black bars) and sorted cKit+ ILCs (n = 1; gray bars).
(TIF)

Table S1 **Immunologically relevant transcripts expressed in cKit+ ILCs categorized by functional category.** All transcripts that met the X^2^ cutoff and were classified in the immunologic functional class are displayed by category along with their relative fold change in cKit+ ILCs.
(XLS)

## References

[1] Neill DR, Wong SH, Bellosi A, Flynn RJ, Daly M, et al (2010) Nuocytes represent a new innate effector leukocyte that mediates type-2 immunity. Nature 464: 1367–1370. 20200518 10.1038/nature08900 PMC2862165

[2] Saenz SA, Siracusa MC, Perrigoue JG, Spencer SP, Urban JF Jr, et al (2010) IL25 elicits a multipotent progenitor cell population that promotes T(H)2 cytokine responses. Nature 464: 1362–1366. 20200520 10.1038/nature08901 PMC2861732

[3] Moro K, Yamada T, Tanabe M, Takeuchi T, Ikawa T, et al (2010) Innate production of T(H)2 cytokines by adipose tissue-associated c-Kit(+)Sca-1(+) lymphoid cells. Nature 463: 540–544. 20023630 10.1038/nature08636

[4] Price AE, Liang HE, Sullivan BM, Reinhardt RL, Eisley CJ, et al (2010) Systemically dispersed innate IL-13-expressing cells in type 2 immunity. Proc Natl Acad Sci U S A 107: 11489–11494. 20534524 10.1073/pnas.1003988107 PMC2895098

[5] Spits H, Artis D, Colonna M, Diefenbach A, Di Santo JP, et al (2013) Innate lymphoid cells–a proposal for uniform nomenclature. Nat Rev Immunol 13: 145–149. 23348417 10.1038/nri3365

[6] Mjosberg JM, Trifari S, Crellin NK, Peters CP, van Drunen CM, et al (2011) Human IL-25- and IL-33-responsive type 2 innate lymphoid cells are defined by expression of CRTH2 and CD161. Nat Immunol 12: 1055–1062. 21909091 10.1038/ni.2104

[7] Bernink JH, Peters CP, Munneke M, te Velde AA, Meijer SL, et al (2013) Human type 1 innate lymphoid cells accumulate in inflamed mucosal tissues. Nat Immunol 14: 221–229. 23334791 10.1038/ni.2534

[8] Roediger B, Kyle R, Yip KH, Sumaria N, Guy TV, et al (2013) Cutaneous immunosurveillance and regulation of inflammation by group 2 innate lymphoid cells. Nat Immunol 14: 564–573. 23603794 10.1038/ni.2584 PMC4282745

[9] Allen JE, Maizels RM (2011) Diversity and dialogue in immunity to helminths. Nat Rev Immunol 11: 375–388. 21610741 10.1038/nri2992

[10] Radermecker M, Bekhti A, Poncelet E, Salmon J (1974) Serum IgE levels in protozoal and helminthic infections. Int Arch Allergy Appl Immunol 47: 285–295. 4854266 10.1159/000231221

[11] King CL (2001) Transmission intensity and human immune responses to lymphatic filariasis. Parasite Immunol 23: 363–371. 11472556 10.1046/j.1365-3024.2001.00395.x

[12] Metenou S, Dembele B, Konate S, Dolo H, Coulibaly SY, et al (2010) At homeostasis filarial infections have expanded adaptive T regulatory but not classical Th2 cells. J Immunol 184: 5375–5382. 20357251 10.4049/jimmunol.0904067 PMC3407820

[13] Nussbaum JC, Van Dyken SJ, von Moltke J, Cheng LE, Mohapatra A, et al (2013) Type 2 innate lymphoid cells control eosinophil homeostasis. Nature. 10.1038/nature12526 PMC3795960 24037376

[14] Di Fiore PP, Scita G (2002) Eps8 in the midst of GTPases. Int J Biochem Cell Biol 34: 1178–1183. 12127568 10.1016/s1357-2725(02)00064-x

[15] Chu PY, Liou JH, Lin YM, Chen CJ, Chen MK, et al (2012) Expression of Eps8 correlates with poor survival in oral squamous cell carcinoma. Asia Pac J Clin Oncol 8: e77–81. 22897151 10.1111/j.1743-7563.2011.01459.x

[16] Yang TP, Chiou HL, Maa MC, Wang CJ (2010) Mithramycin inhibits human epithelial carcinoma cell proliferation and migration involving downregulation of Eps8 expression. Chem Biol Interact 183: 181–186. 19799886 10.1016/j.cbi.2009.09.018

[17] Khan AP, Contessa JN, Nyati MK, Ross BD, Rehemtulla A (2011) Molecular imaging of epidermal growth factor receptor kinase activity. Anal Biochem 417: 57–64. 21693098 10.1016/j.ab.2011.05.040 PMC3204941

[18] Ivaska J, Heino J (2010) Interplay between cell adhesion and growth factor receptors: from the plasma membrane to the endosomes. Cell Tissue Res 339: 111–120. 19722108 10.1007/s00441-009-0857-z PMC2784865

[19] Xu Z, Xiao SB, Xu P, Xie Q, Cao L, et al (2011) miR-365, a novel negative regulator of interleukin-6 gene expression, is cooperatively regulated by Sp1 and NF-kappaB. J Biol Chem 286: 21401–21412. 21518763 10.1074/jbc.M110.198630 PMC3122200

[20] Semnani RT, Venugopal PG, Leifer CA, Mostbock S, Sabzevari H, et al (2008) Inhibition of TLR3 and TLR4 function and expression in human dendritic cells by helminth parasites. Blood 112: 1290–1298. 18541719 10.1182/blood-2008-04-149856 PMC2515123

[21] Semnani RT (2013) The interaction between filarial parasites and human monocyte/macrophage populations. Adv Exp Med Biol 785: 49–56. 23456837 10.1007/978-1-4614-6217-0_6

[22] Segura E, Touzot M, Bohineust A, Cappuccio A, Chiocchia G, et al (2013) Human inflammatory dendritic cells induce Th17 cell differentiation. Immunity 38: 336–348. 23352235 10.1016/j.immuni.2012.10.018

[23] Licona-Limon P, Kim LK, Palm NW, Flavell RA (2013) TH2, allergy and group 2 innate lymphoid cells. Nat Immunol 14: 536–542. 23685824 10.1038/ni.2617

[24] Palm NW, Rosenstein RK, Yu S, Schenten DD, Florsheim E, et al (2013) Bee venom phospholipase A2 induces a primary type 2 response that is dependent on the receptor ST2 and confers protective immunity. Immunity 39: 976–985. 24210353 10.1016/j.immuni.2013.10.006 PMC3852615

[25] Abdelmohsen K, Srikantan S, Tominaga K, Kang MJ, Yaniv Y, et al (2012) Growth inhibition by miR-519 via multiple p21-inducing pathways. Mol Cell Biol 32: 2530–2548. 22547681 10.1128/MCB.00510-12 PMC3434494

[26] Lin J, Huang S, Wu S, Ding J, Zhao Y, et al (2011) MicroRNA-423 promotes cell growth and regulates G(1)/S transition by targeting p21Cip1/Waf1 in hepatocellular carcinoma. Carcinogenesis 32: 1641–1647. 21890460 10.1093/carcin/bgr199

[27] Keklikoglou I, Koerner C, Schmidt C, Zhang JD, Heckmann D, et al (2012) MicroRNA-520/373 family functions as a tumor suppressor in estrogen receptor negative breast cancer by targeting NF-kappaB and TGF-beta signaling pathways. Oncogene 31: 4150–4163. 22158050 10.1038/onc.2011.571

[28] Spooner CJ, Lesch J, Yan D, Khan AA, Abbas A, et al (2013) Specification of type 2 innate lymphocytes by the transcriptional determinant Gfi1. Nat Immunol. 10.1038/ni.2743 24141388

[29] Gentek R, Munneke JM, Helbig C, Blom B, Hazenberg MD, et al (2013) Modulation of Signal Strength Switches Notch from an Inducer of T Cells to an Inducer of ILC2. Front Immunol 4: 334. 24155745 10.3389/fimmu.2013.00334 PMC3804867

