# Supplementary figures and images for: Human CD117 (cKit)+ Innate Lymphoid Cells Have a Discrete Transcriptional Profile at Homeostasis and Are Expanded during Filarial Infection
Source: PLoS One. 2014 Sep 25;9(9):e108649. doi: 10.1371/journal.pone.0108649 (PMC4177898; doi:10.1371/journal.pone.0108649)

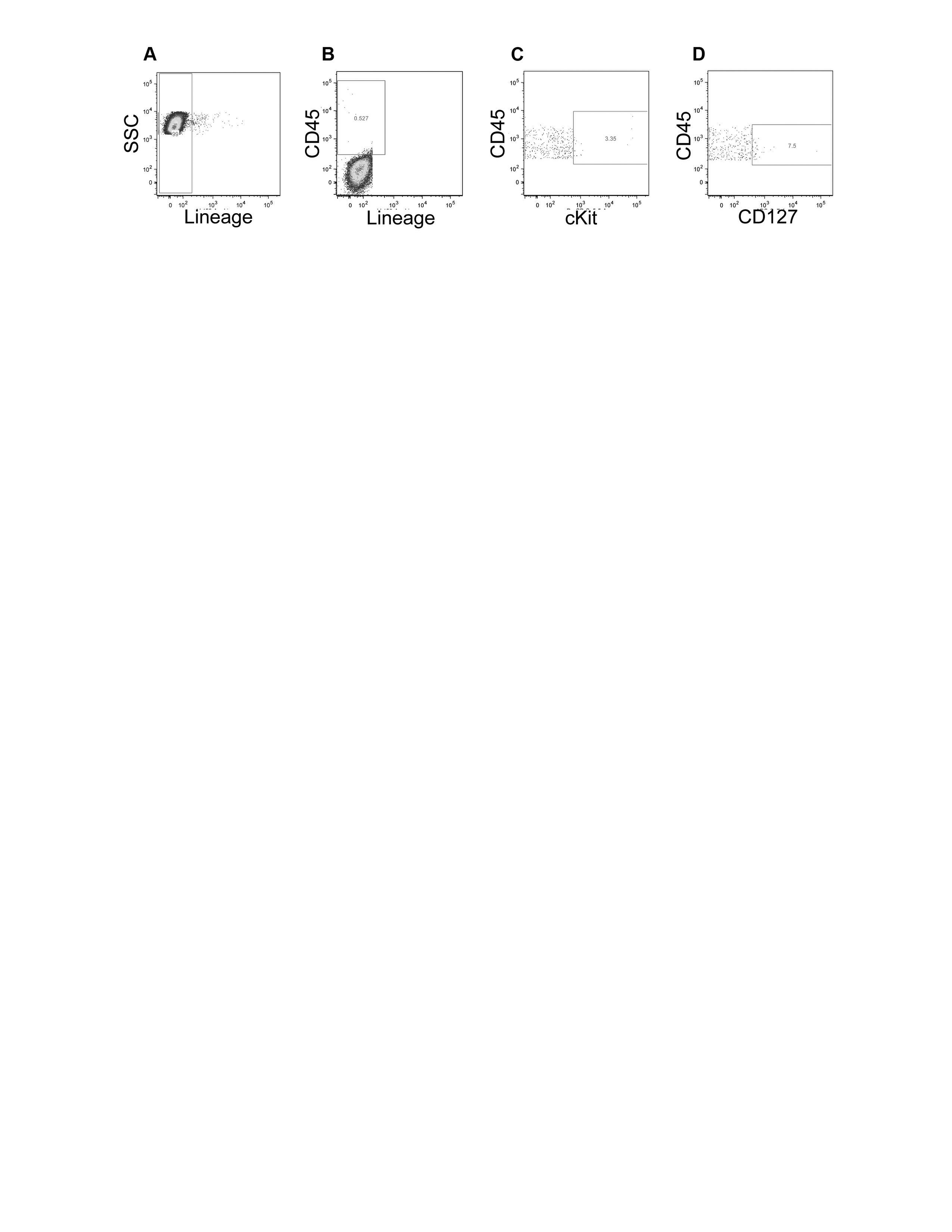

Supplement: Figure S1 — Fluorescence minus one controls for ILC flow cytometry gating strategy. Fluorescence minus one (FMO) controls were obtained by adding all but one antibody stain to PBMCs isolated from normal patients for (A) the Lineage panel, (B) CD45, (C) cKit and (D) CD127. (TIF) [file pone.0108649.s001.tif]

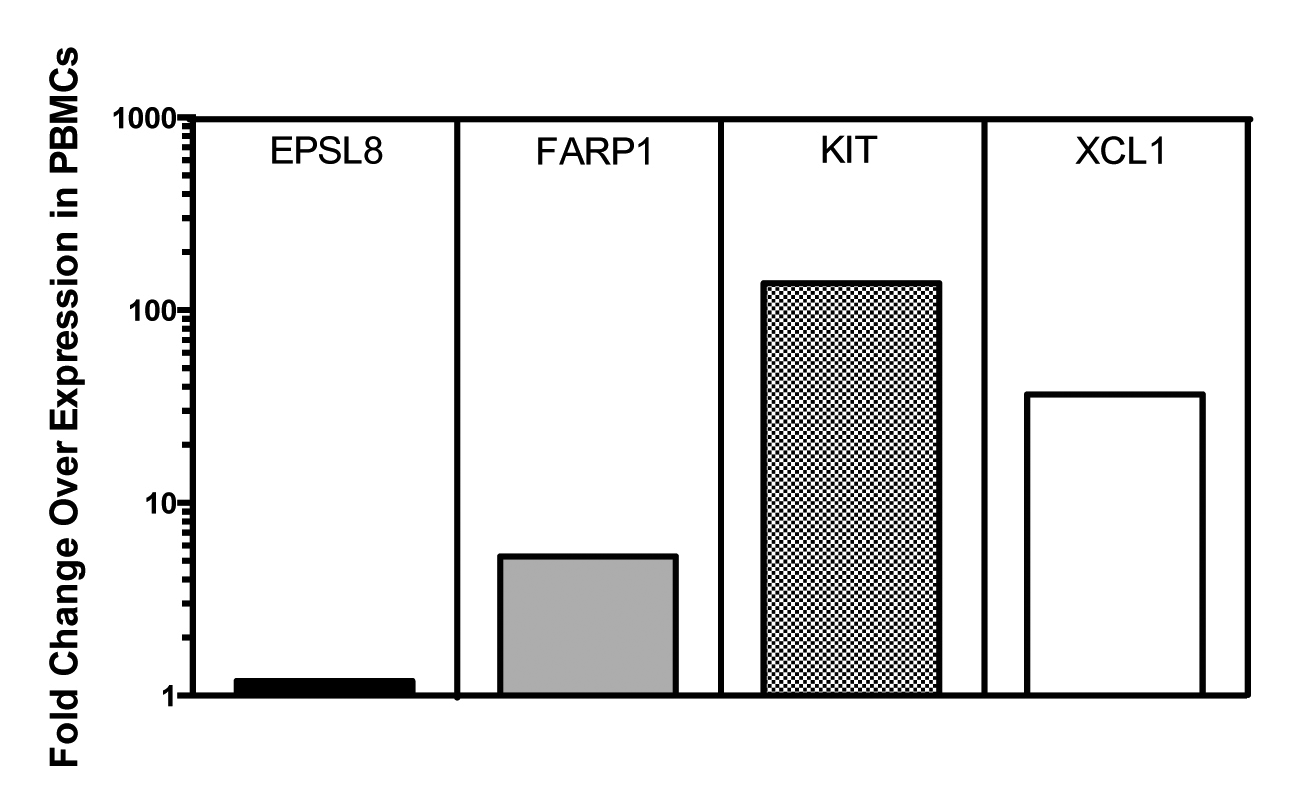

Supplement: Figure S2 — Top upregulated transcripts in the cKit+ ILCs transcriptome are confirmed with RT-PCR. RNA was extracted from cryopreserved PBMCs and sorted cKit+ ILCs from normal donors; mRNA levels were measured by real-time PCR and normalized to the levels of 18S ribosomal RNA. Results are shown as 1/ΔCT for normal PBMCs (n = 1; black bars) and sorted cKit+ ILCs (n = 1; gray bars). (TIF) [file pone.0108649.s002.tif]
